# Diagnostic scope: the AI can’t see what the mind doesn’t know

**DOI:** 10.1515/dx-2024-0151

**Published:** 2024-12-04

**Authors:** Gary E. Weissman, Laura Zwaan, Sigall K. Bell

**Affiliations:** Palliative and Advanced Illness Research (PAIR) Center, University of Pennsylvania Perelman School of Medicine, Philadelphia, PA, USA; Pulmonary, Allergy, and Critical Care Division, Department of Medicine, University of Pennsylvania Perelman School of Medicine, Philadelphia, PA, USA; Division of Informatics, Department of Biostatistics, Epidemiology & Informatics, University of Pennsylvania Perelman School of Medicine, Philadelphia, PA, USA; and Leonard Davis Institute of Health Economics, University of Pennsylvania, Philadelphia, PA, USA; Institute of Medical Education Research, Erasmus Medical Center, Rotterdam, The Netherlands.; Department of Medicine, Beth Israel Deaconess Medical Center, Harvard Medical School, Boston, MA, USA

**Keywords:** diagnostic scope, artificial intelligence, human-AI collaboration, diagnostic reasoning

## Abstract

**Background:**

Diagnostic scope is the range of diagnoses found in a clinical setting. Although the diagnostic scope is an essential feature of training and evaluating artificial intelligence (AI) systems to promote diagnostic excellence, its impact on AI systems and the diagnostic process remains under-explored.

**Content:**

We define the concept of diagnostic scope, discuss its nuanced role in building safe and effective AI-based diagnostic decision support systems, review current challenges to measurement and use, and highlight knowledge gaps for future research.

**Summary:**

The diagnostic scope parallels the differential diagnosis although the latter is at the level of an encounter and the former is at the level of a clinical setting. Therefore, diagnostic scope will vary by local characteristics including geography, population, and resources. The true, observed, and considered scope in each setting may also diverge, both posing challenges for clinicians, patients, and AI developers, while also highlighting opportunities to improve safety. Further work is needed to systematically define and measure diagnostic scope in terms that are accurate, equitable, and meaningful at the bedside. AI tools tailored to a particular setting, such as a primary care clinic or intensive care unit, will each require specifying and measuring the appropriate diagnostic scope.

**Outlook:**

AI tools will promote diagnostic excellence if they are aligned with patient and clinician needs and trained on an accurately measured diagnostic scope. A careful understanding and rigorous evaluation of the diagnostic scope in each clinical setting will promote optimal care through human-AI collaborations in the diagnostic process.

## Introduction

When a patient presents with an uncertain constellation of signs and symptoms, a thoughtful clinician considers a broad range of potential etiologies in the differential diagnosis. To achieve an accurate and timely explanation for the underlying problem, the cause of these symptoms is hopefully contained within that list. Appropriately, most efforts to promote diagnostic excellence have focused on the diagnostic reasoning process at the level of a single clinical encounter. By contrast, the diagnostic scope is the aggregation of diagnoses over all encounters at the level of a geographic region, practice setting, or clinical specialty. However, relatively less attention has been paid to this critical feature of the diagnostic process. Additionally, no universally agreed-upon definition of diagnostic scope exists. Yet, the importance of diagnostic scope is actively growing as the potential for artificial intelligence (AI) and machine learning (ML) tools are being used in diagnostic clinical decision support systems (CDSSs). The diagnostic scope informs the development and training of diagnostic AI/ML CDSSs and therefore must be accurately specified in order for those systems to be useful in their desired context.

The importance of diagnostic scope for AI/ML CDSS development arises because of how such systems are trained. Predictive CDSSs typically require humans to specify the outcomes for which the computational models will learn patterns in the training data. For example, a data scientist building an AI/ML diagnostic CDSS for an infectious disease clinic would have to know ahead of time every diagnosis that might be encountered in a given practice setting. But characterizing this broad range of potential diagnoses is not straightforward. Humans, let alone computers, have yet to devise a system of diagnostic categories that are both readily analyzable in large clinical datasets and also useful for clinical decision making at the bedside. Thus, optimal human-AI collaboration for diagnostic excellence will require developing a shared language to describe the diagnostic scope. Patients, too, have a critical role to play in defining the diagnostic scope and setting priorities for its development.

Accurate assessments of diagnostic scope will foster efforts to enhance patient safety, optimize staffing models and clinic organization, educate clinicians in diagnostic reasoning, and improve the diagnostic process for patients and clinicians. In this Viewpoint, we review the current literature on diagnostic scope, propose a standardized definition, consider nuances in its measurement, and highlight critical challenges and areas for further research to realize the potential of AI/ML CDSSs to promote diagnostic excellence.

## Defining diagnostic scope

**Figure 1:**
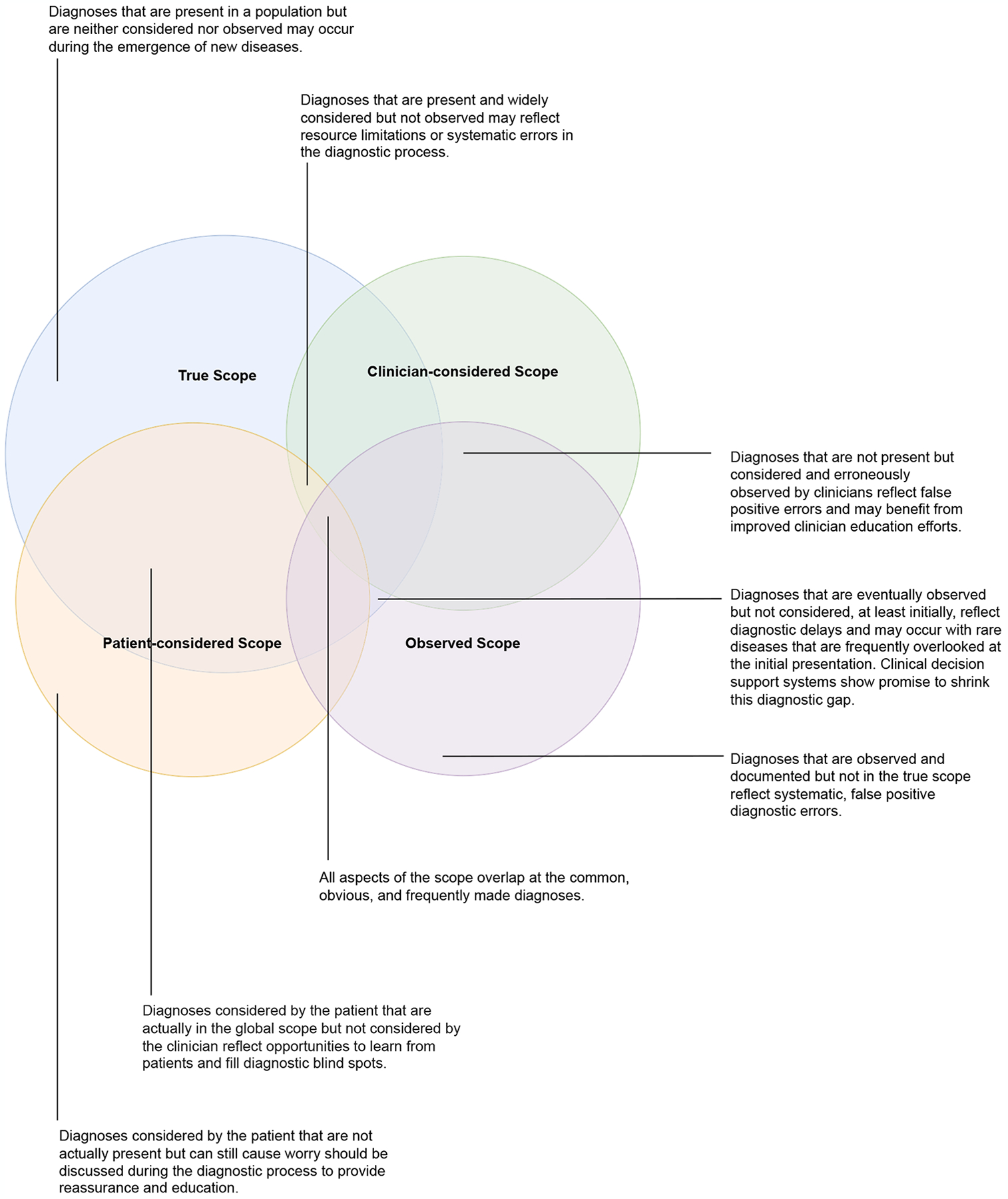
The true diagnostic scope includes the full range of diagnoses present in a particular clinical setting. Patients and clinicians in a clinical setting each may consider a distinct range of diagnoses. The observed diagnostic scope reflects all documented diagnoses, right or wrong, in a specific setting. Each aspect of the diagnostic scope both overlaps and diverges from the others. Importantly, the figure is not drawn to any particular scale.

We propose that the true diagnostic scope should be defined as a theoretical construct that includes all possible diagnoses in a given setting (Figure 1). The considered scopes reflect those diagnoses that clinicians and patients entertain during the diagnostic journey. And the observed scope reflects those diagnoses, correct or not, that are documented as present. Each of these scopes may overlap with and diverge from each other. For example, there may be a gap between the considered and observed scopes if some diagnoses are frequently included in the differential diagnosis or tested for but not actually made. Conversely, there may be some diagnoses that are not considered, but should have been, and are therefore still present in a population but not observed. This latter category includes over 10,000 rare diseases, or “zebras” that are frequently missed during the diagnostic process [1, 2]. Rare conditions such as inherited metabolic disorders or certain neoplasms constitute an important element of the true scope that may not be considered by clinicians. These diagnoses reflect false negative diagnostic errors. Each distinct and overlapping area of clinician-considered, patient-considered, observed, and true diagnostic scope offers unique opportunities to improve patient safety and the diagnostic process (Figure 1).

Similarly, there is a distinction between the range of diagnoses that are possible and those that are likely. Therefore, a useful AI/ML CDSS will not only account for the range of diagnoses, but also the probability of each one. Thus, an accurate weighting of the true scope will support the diagnostic process because not every diagnosis in the true scope is equally likely to present during any individual encounter. However, this probability distribution will also likely vary by the practice setting due to heterogeneity in geographic or population-based disease prevalence.

The diagnostic scope has been described to varying extents in previous studies. Prior work has quantified geographic and specialty-specific variation in the content and breadth of documented diagnoses but has not accounted for weighting of the diagnostic scope by disease prevalence [3–7]. Nor have any previous studies distinguished between true, considered, and observed scopes. In a distinct but related sense of the term, the diagnostic scope has also referred to the range of conditions that a particular test might identify [8, 9]. In this sense, each AI/ML diagnostic CDSS system might analogously have its own diagnostic scope, aligned or not, with the true scope of the clinical setting in which it is used.

## Dependence on local factors

The diagnostic scope in any given setting is dependent on local features of the population, including the age distribution, socioeconomic factors, environmental exposures, and endemicity of diseases. For example, one study of Medicare claims identified strong similarities between diagnostic scopes in rural and urban practices but also found large differences between specialty-specific settings, such as between obstetrician-gynecology and general surgery practices [4].

Access to care and referral patterns related to local healthcare infrastructure may also shape the observed diagnostic scope since clinicians may not identify conditions in patients who do not present to care. This presents a critical risk of systemic bias in data. Similarly, clinicians may disproportionately encounter early, presenting symptoms of a disease process in one setting (or among a particular population that is more likely to seek early care) that only later manifest fully with a diagnosis made in a different setting. This information gap is well known for clinicians who often lack diagnostic feedback about their decisions [10] and poses a related threat to accurately measuring the diagnostic scope. Moreover, the availability of resources to detect diseases may also contribute to the extent to which the considered and observed scopes diverge.

For example, lack of access to molecular testing is a key driver of the “diagnostic gap” in identifying tuberculosis [11].

Local differences also underscore the need for AI/ML systems that are trained and validated with data relevant to the populations for which they will be used. For example, while gallstones and alcohol use are the most common causes of pancreatitis worldwide, parasitic infection has been identified as the etiology in nearly a quarter of cases in some parts of the world with ascaris endemicity [12], and should therefore be considered in a useful diagnostic CDS in these settings. However, it likely falls outside the considered scope in most parts of the US and may be absent entirely from the observed scope, and thus also from the training data used for a US-based AI/ML CDSS. Even if the scope is perfectly defined in a given setting, and an AI/ML CDSS is trained with accurate, relevant data to predict that scope, the performance of the model will depend on where and when it is used. Thus, the diagnostic scope should be assessed for external validity or spectrum bias, analogous concept from the literature on predictive modeling and diagnostic testing, in which a tool’s accuracy may change in a population different from the one used for its development [13, 14]. Finally, only prospective evaluations of clinical effectiveness [15] will reliably estimate the accuracy of an AI/ML model’s scope in a given context. By contrast, *in silico* evaluations, or those that only measure a model’s predictive performance against outcomes in a retrospectively collected dataset [16], are insufficient to identify an AI/ML model trained on a misspecified diagnostic scope.

## Clinician-AI diagnostic reasoning

Clinicians often rely on a range of heuristics in decision making when faced with uncertainty [17, 18] and AI/ML CDSSs show promise to support such decisions. However, there is a circular relationship between the clinicians’ work of observing and documenting diagnosis and the training data that AI/ML models will use to provide decision support back to clinicians, potentially amplifying systemic bias and inaccurate diagnoses. Clinicians may miss important diagnoses if they are not frequently observed [19], leading AI/ML CDSSs to do the same.

Conversely, false positive diagnoses may occur if some diseases are considered in scope due to saliency or availability bias but infrequently observed. Again, AI/ML CDSSs will learn to make the same diagnostic errors. This risk may be further exacerbated by clinicians who insufficiently review the basis for recommendations from an AI/ML CDSS and follow them anyway. This phenomenon, known as “automation bias” is a key safety threat when using AI/ML CDSSs [20, 21].

Diagnostic AI/ML CDSSs, if trained on an accurately weighted diagnostic scope, are especially promising adjuncts to human clinicians during the diagnostic reasoning process. For example, a binary assessment of in/out of scope along with a quantitative assessments of a disease’s prevalence may guide decision making. This information could help to reduce cognitive errors due to the base rate fallacy (the tendency for a clinician to ignore the known prevalence of a disease in a population when estimating the probability of that disease in a patient) [22]. A nuanced framing of diagnostic scope and the interactions between types of scope (Figure 1) may prove useful in medical education to facilitate diagnostic reasoning and history elicitation. As AI/ML technologies are increasingly evaluated for their diagnostic capabilities and integrated into the clinical curriculum [23–25], the next generation of clinicians will require a sophisticated understanding of these concepts and the risk of circular relationships to maximize human-AI cooperation and accuracy in the diagnostic process.

For example, an AI/ML CDSS may not be useful during a pandemic in which a new disease becomes common if the CDSS was trained prior to the pandemic. In this case, the disease may not even have been in the observed scope reflected in the training data. Or it may have been in scope but much less prevalent. In either case, following the suggestions of this AI/ML CDSS during a pandemic would likely lead to false negative errors, or missed diagnoses. Automation bias would exacerbate this pattern and lead to further missed diagnoses. Even if the underlying AI/ML model were updated to reflect pandemic conditions, it may still lead to under-diagnosis by learning, and then recapitulating, the patterns of missed diagnoses early in the pandemic. For such AI/ML CDSSs to be effective at the bedside, they must reflect not only geographic but also temporal changes in the diagnostic scope [26]. And clinicians must remain vigilant to maintain their own independent cognition during the diagnostic process while simultaneously accounting for the limitations of a particular AI/ML system.

## Learning from patients

While generally considered the purview of clinicians, development and assessment of diagnostic scope should also incorporate the unique knowledge and experiences of patients. Currently, a diagnostic error is defined as either failing to identify the correct diagnosis in a timely fashion or failing to communicate that diagnosis to a patient [27]. But this definition hinges only on the final documented diagnosis and does not account for any diagnoses that were considered during the diagnostic process, on any contributions of the patient to that process, or on patients’ knowledge of inaccurate diagnoses that were subsequently corrected at another healthcare center. Patients themselves may contribute important experiences and insights during the diagnostic journey that can inform not only clinician learning, but also organizational culture and practices that minimize communication breakdowns and promote patient- and family-centered care [28].

The model of diagnosis as “wayfinding” incorporates iterative information gathering, deliberation, and integration of insights from both patients and clinicians to eventually arrive at a correct diagnosis [29]. Listening closely to patients and their stories [30] can help identify elements of the true scope not otherwise captured in the clinician’s considered scope. A distributed cognition model that accounts for regions of the patient-considered scope that are outside of the clinician-considered scope can help to avoid diagnostic blind spots [31] (Figure 1). Taking the next step to proactively elicit, aggregate, and learn from patient experiences of diagnostic breakdowns can also enrich clinicians’ considered or observed scope, with the potential to reduce systemic bias and improve diagnostic accuracy and safety, especially if feedback systems “close the loop” with clinicians involved earlier in the diagnostic process [32–35].

Symptom-focused internet searches are increasingly common and lead patients to consider their own differential diagnoses. In aggregate, and coupled with the patients’ own lived experiences, these may inform the patient-considered diagnostic scope (Figure 1). Patients are increasingly using AI for self-diagnosis. If patient and clinician AI/ML tools are trained on data with differing diagnostic scope, the tools may generate misalignments between patient and clinician diagnostic considerations. While a patient may be more likely to trust their clinician than an AI/ML CDSS, this preference may shift over time or with clinicians that patients do not know well or do not trust, potentially undermining the patient-clinician relationship [36]. Similar to the guidance for clinicians to share trustworthy internet sites with patients, organizations and clinicians may consider guiding patients to context-specific AI/ML resources. Healthcare organizations and individual clinicians will likely view the transparent development and reporting of the diagnostic scope of AI/ML CDSSs as assets in considering these recommendations.

Patient preferences should also inform prioritization of diagnostic scope research and quality improvement efforts. For example, benign, self-limited rashes may have high diagnostic uncertainty and yet may not be frequently represented in the observed scope because a true diagnosis is not definitively made. But such diagnoses likely produce little direct patient harm. By contrast, other diagnoses that are frequently missed or delayed despite their representation in the diagnostic scope, such as heart failure, urinary tract infections, and cancer, can result in significant harm and should receive priority [37–40].

## Measuring diagnostic scope

One reason that diagnostic scope may be understudied is that it is challenging to measure. A lack of open-source and clinically relevant ontologies has so far limited empiric evaluation. For example, a large study of primary care encounters across three countries used a proprietary mapping of diagnostic codes to clinically relevant clusters [5]. Such proprietary systems have limited reproducibility and transparency which preclude their widespread evaluation and validation in clinical practice. On the other hand, several freely available diagnostic ontologies are widely studied and easy to integrate into data analysis pipelines. These include the 61,048 Medical Subject Headings (MeSH) unique concepts as of 2024 [41] and the 71,932 International Classification of Diseases (ICD) - 10 codes [42]. However, such ontologies are so broad and specific as to be clunky at the bedside for clinicians and patients engaged in the diagnostic process. Thus, for purposes of measuring diagnostic scope for building AI/ML models, quality improvement efforts, or medical education, the optimal level of granularity and which diagnostic categories are most useful remain unknown. Finally, the validity of any reported diagnostic scope in practice will depend, to some extent, on both the methods and data sources used to measure it.

## Promise and pitfalls of AI/ML tools

Once the relevant diagnostic scope is identified and measured for a given clinical setting, an AI/ML CDSS can be trained to identify patient factors that predict individual diagnoses. While AI/ML CDSSs offer promise to improve the diagnostic process through the ability to learn patterns in massive datasets and generate personalized recommendations, there are several ways in which an AI/ML model trained on an incorrectly specified diagnostic scope may exacerbate existing health disparities. Because AI/ML systems are built for scalability, they risk amplifying the errors in diagnostic reasoning discussed above. For example, acute coronary syndromes are much more frequently misdiagnosed in women compared to men. If an erroneous inclusion or weighting of such diseases in the diagnostic scope is used to train an AI/ML CDSS, that system will invariably reinforce the same diagnostic errors in practice that led to misdiagnosis in the first place [43, 44]. An AI/ML CDSS thus create an opportunity for the errors made by clinicians whose actions were captured in the training data to propagate to new settings and encounters. AI/ML approaches increase this risk, especially when the observed diagnostic scope overlooks sickle cell disease [45], malaria [46], cystic fibrosis [47], and other rare diseases [48].

At the same time, such systems also offer opportunities to mitigate disparities and the potential harmful effects of interpersonal bias, prejudice, or incomplete medical knowledge. Because most diagnostic errors are due to suboptimal history taking or physical examination [37], a properly trained AI/ML system could help to facilitate the diagnostic process and improve diagnostic accuracy by filling cognitive gaps or mitigating biases. These systems may be further strengthened by focused research to identify missed or delayed diagnoses in marginalized populations, considering access to care and delayed presentations and other intersectional factors such as English language proficiency and socio-economic disparities alongside clinician bias. In each of these cases, the diagnostic AI/ML system will only be as good as the data on which it is trained, which in turn, is created, curated, and chosen by humans, requiring principled approaches and multidisciplinary oversight [49, 50].

## Tensions, challenges, and opportunities for diagnostic scope

The importance of diagnostic scope is increasingly recognized, especially in the context of developing AI/ML CDSSs to improve the diagnostic process. But several obstacles must be overcome before it becomes directly useful to researchers, clinicians, and patients. First, a pragmatic application of the true scope, weighted by both prevalence and clinical importance, must be identified for each setting. While almost all diagnoses *could* be present in most clinical contexts, those with infinitesimal prevalence are unlikely to be useful in developing diagnostic AI/ML CDSSs unless their clinical importance outweighs their rarity. For example, Ebola virus disease has temporally sporadic and geographically heterogeneous prevalence – even approaching zero in some locations – but its early diagnosis remains a key global health priority [51]. Further work is needed to better characterize how the diagnostic scope is sensitive to local factors and how it may vary in both content and breadth. Second, more work is needed to develop clinically meaningful diagnostic ontologies that will be useful at the bedside. A useful ontology will balance the simultaneous needs for generalizability and specificity. Third, improved measurement tools and data sharing policies will be needed to ensure accurate assessments of true, considered, and observed scopes. Transparency, interoperability, and protection of patient privacy are all necessary for successful measurement and analysis of diagnostic scope. Fourth, evolving governance frameworks for AI/ML CDSSs will need to ensure clinical effectiveness, safety, and equity. Only in such a regulatory environment will the benefits of future research efforts into diagnostic scope be realized.

In summary, the diagnostic scope is a key feature of the diagnostic process and will play an increasingly vital role with rising uptake of AI/ML CDSSs in healthcare, but it remains largely under-explored. More research is needed to better refine and quantify its role in clinical, cognitive, policy, and analytic contexts to improve patient care and outcomes through diagnostic excellence. Just as “the eye can’t see what the mind doesn’t know,” so too an AI/ML model can’t predict what the humans don’t tell it.

## Data Availability

Not applicable.

## References

[1] Haendel M, Vasilevsky N, Unni D, Bologa C, Harris N, Rehm H, How many rare diseases are there? Nat Rev Drug Discov 2020;19:77–8. 32020066 10.1038/d41573-019-00180-y PMC7771654

[2] Gold JI. When the hoofbeats are zebras. Ann Intern Med 2022;1:e220352C.

[3] Draye MA, Pesznecker BL. Diagnostic scope and certainty: an analysis of FNP practice. Nurse Pract 1979;4:42–3, passim. 759998

[4] Baldwin LM, Rosenblatt RA, Schneeweiss R, Lishner DM, Hart LG. Rural and urban physicians: does the content of their Medicare practices differ? J Rural Health 1999;15:240–51. 10511761 10.1111/j.1748-0361.1999.tb00745.x

[5] Bindman AB, Forrest CB, Britt H, Crampton P, Majeed A. Diagnostic scope of and exposure to primary care physicians in Australia, New Zealand, and the United States: cross sectional analysis of results from three national surveys. BMJ 2007;334:1261–2. 17504790 10.1136/bmj.39203.658970.55 PMC1892467

[6] Huibers LA, Moth G, Bondevik GT, Kersnik J, Huber CA, Christensen MB, Diagnostic scope in out-of-hours primary care services in eight European countries: an observational study. BMC Family Practice 2011;12:30–1. 21569483 10.1186/1471-2296-12-30 PMC3114765

[7] Moth G, Huibers L, Christensen MB, Vedsted P. Out-of-Hours primary care: a population- based study of the diagnostic scope of telephone contacts. Fam Pract 2016;33:504–9. 27328678 10.1093/fampra/cmw048

[8] Shin H, Choi BH, Shim O, Kim J, Park Y, Cho SK, Single test-based diagnosis of multiple cancer types using exosome-SERS-AI for early stage cancers. Nat Commun 2023;14:1644–5. 36964142 10.1038/s41467-023-37403-1 PMC10039041

[9] Prager R, Bowdridge J, Pratte M, Cheng J, McInnes MD, Arntfield R. Indications, clinical impact, and complications of critical care transesophageal echocardiography: a scoping review. J Intensive Care Med 2023;38:245–72. 35854414 10.1177/08850666221115348 PMC9806486

[10] Fernandez Branson C, Williams M, Chan TM, Graber ML, Lane KP, Grieser S, Improving diagnostic performance through feedback: the diagnosis learning cycle. BMJ Qual Saf 2021;30:1002–9. 10.1136/bmjqs-2020-012456 PMC8606468 34417335

[11] Pai M, Dewan PK, Swaminathan S. Transforming tuberculosis diagnosis. Nat Microbiol 2023;8:756–9. 37127703 10.1038/s41564-023-01365-3

[12] Khuroo MS, Rather AA, Khuroo NS, Khuroo MS. Hepatobiliary and pancreatic ascariasis. World J Gastroenterol 2016;22:7507–17. 27672273 10.3748/wjg.v22.i33.7507 PMC5011666

[13] Riley RD, Archer L, Snell KIE, Ensor J, Dhiman P, Martin GP, Evaluation of clinical prediction models (Part 2): how to undertake an external validation study. BMJ 2024;384:e74820. 10.1136/bmj-2023-074820 PMC10788734 38224968

[14] Goehring C, Perrier A, Morabia A. Spectrum bias: a quantitative and graphical analysis of the variability of medical diagnostic test performance. Stat Med 2004;23:125–35. 14695644 10.1002/sim.1591

[15] Longhurst CA, Singh K, Chopra A, Atreja A, Brownstein JS. A call for artificial intelligence implementation science centers to evaluate clinical effectiveness. NEJM AI 2024;0:AIp2400223.

[16] Weissman GE. Moving from in silico to in clinico evaluations of machine learning-based interventions in critical care. Crit Care Med 2024;52:1141–4. 38869387 10.1097/CCM.0000000000006277

[17] Tversky A, Kahneman D. Judgment under uncertainty: heuristics and biases. Science 1974;185:1124–31. 17835457 10.1126/science.185.4157.1124

[18] Croskerry P. The importance of cognitive errors in diagnosis and strategies to minimize them. Acad Med 2003;78:775–80. 12915363 10.1097/00001888-200308000-00003

[19] Evans KK, Birdwell RL, Wolfe JM. If you don’t find it often, you often don’t find it: why some cancers are missed in breast cancer screening. PLoS One 2013;8:e64366. 23737980 10.1371/journal.pone.0064366 PMC3667799

[20] Dratsch T, Chen X, Rezazade Mehrizi M, Kloeckner R, Mähringer-Kunz A, Püsken M, Automation bias in mammography: the impact of artificial intelligence BI-RADS suggestions on reader performance. Radiology 2023;307:222176–7. 10.1148/radiol.222176 37129490

[21] Jabbour S, Fouhey D, Shepard S, Valley TS, Kazerooni EA, Banovic N, Measuring the impact of AI in the diagnosis of hospitalized patients: a randomized clinical vignette survey study. JAMA 2023;330:2275–84. 38112814 10.1001/jama.2023.22295 PMC10731487

[22] Bar-Hillel M. The base-rate fallacy in probability judgments. Acta Psychologica 1980;44:211–33.

[23] Cabral S, Restrepo D, Kanjee Z, Wilson P, Crowe B, Abdulnour R-E, Clinical reasoning of a generative artificial intelligence model compared with physicians. JAMA Intern Med 2024;184:581–3. 38557971 10.1001/jamainternmed.2024.0295 PMC10985627

[24] Weidener L, Fischer M. Artificial intelligence teaching as part of medical education: qualitative analysis of expert interviews. JMIR Med Educ 2023;9:e46428. 36946094 10.2196/46428 PMC10167581

[25] Russell RG, Lovett Novak L, Patel M, Garvey KV, Craig KJT, Jackson GP, Competencies for the use of artificial intelligence–based tools by health care professionals. Acad Med 2023;98:348–9. 36731054 10.1097/ACM.0000000000004963

[26] Mousavi SM, Alghisi S, Riccardi G. DyKnow: dynamically verifying time-sensitive factual knowledge in LLMs. In: Al-Onaizan Y, Bansal M, Chen Y-N, editors. Findings of the association for computational linguistics: EMNLP 2024. Miami, Florida, USA: Association for Computational Linguistics; 2024:8014–29 pp.

[27] Committee on Diagnostic Error in Health Care, Board on Health Care Services, Institute of Medicine, The National Academies of Sciences, Engineering, and Medicine. Balogh EP, Miller BT, Ball JR, editors. Improving diagnosis in health care. Washington, D.C.: National Academies Press; 2015:21794–5 pp. 26803862

[28] Bell SK, Bourgeois F, DesRoches CM, Dong J, Harcourt K, Liu SK, Filling a gap in safety metrics: development of a patient-centred framework to identify and categorise patient- reported breakdowns related to the diagnostic process in ambulatory care. BMJ Qual Saf 2022;31:526–40. 10.1136/bmjqs-2021-013672 34656982

[29] Adler-Milstein J, Chen JH, Dhaliwal G. Next-generation artificial intelligence for diagnosis: from predicting diagnostic labels to “wayfinding”. JAMA 2021;326:2467–8. 34882190 10.1001/jama.2021.22396 PMC12049696

[30] Lee YSH, Grob R, Nembhard I, Shaller D, Schlesinger M. Leveraging patients’ creative ideas for innovation in health care. Milbank Q 2024;102:233–69. 38090879 10.1111/1468-0009.12682 PMC10938936

[31] Bell SK, Bourgeois F, Dong J, Gillespie A, Ngo LH, Reader TW, Patient identification of diagnostic safety blindspots and participation in “good catches” through shared visit notes. Milbank Q 2022;100:1121–65. 36539389 10.1111/1468-0009.12593 PMC9836247

[32] Giardina TD, Haskell H, Menon S, Hallisy J, Southwick FS, Sarkar U, Learning from patients’ experiences related to diagnostic errors is essential for progress in patient safety. Health Aff 2018;37:1821–7. 10.1377/hlthaff.2018.0698 PMC8103734 30395513

[33] Bell SK, Dong J, Ngo L, McGaffigan P, Thomas EJ, Bourgeois F. Diagnostic error experiences of patients and families with limited English-language health literacy or disadvantaged socioeconomic position in a cross-sectional US population-based survey. BMJ Qual Saf 2023;32:644–54. 10.1136/bmjqs-2021-013937 35121653

[34] Bell SK, Harcourt K, Dong J, DesRoches C, Hart NJ, Liu SK, Patient and family contributions to improve the diagnostic process through the OurDX electronic health record tool: a mixed method analysis. BMJ Qual Saf 2024;33:597–608. 10.1136/bmjqs-2022-015793 PMC10879445 37604678

[35] Bell SK, Dong ZJ, Desroches CM, Hart N, Liu S, Mahon B, Partnering with patients and families living with chronic conditions to coproduce diagnostic safety through OurDX: a previsit online engagement tool. J Am Med Inf Assoc 2023;30:692–702. 10.1093/jamia/ocad003 PMC10018262 36692204

[36] Zondag AGM, Rozestraten R, Grimmelikhuijsen SG, Jongsma KR, van Solinge WW, Bots ML, The effect of artificial intelligence on patient-physician trust: cross-sectional vignette study. J Med Internet Res 2024;26:e50853. 38805702 10.2196/50853 PMC11167322

[37] Singh H, Giardina TD, Meyer AND, Forjuoh SN, Reis MD, Thomas EJ. Types and origins of diagnostic errors in primary care settings. JAMA Intern Med 2013;173:418–9. 23440149 10.1001/jamainternmed.2013.2777 PMC3690001

[38] Singh H, Meyer AND, Thomas EJ. The frequency of diagnostic errors in outpatient care: estimations from three large observational studies involving US adult populations. BMJ Qual Saf 2014;23:727–31. 10.1136/bmjqs-2013-002627 PMC4145460 24742777

[39] Gandhi TK, Kachalia A, Thomas EJ, Puopolo AL, Yoon C, Brennan TA, Missed and delayed diagnoses in the ambulatory setting: a study of closed malpractice claims. Ann Intern Med 2006;145:488–9. 17015866 10.7326/0003-4819-145-7-200610030-00006

[40] Saber Tehrani AS, Lee H, Mathews SC, Shore A, Makary MA, Pronovost PJ, 25-Year summary of US malpractice claims for diagnostic errors 1986–2010: an analysis from the national practitioner data bank. BMJ Qual Saf 2013;22:672–80. 10.1136/bmjqs-2012-001550 23610443

[41] Rogers FB. Communications to the Editor. Bull Med Libr Assoc 1963;51:114–6. 13982385 PMC197951

[42] Utter GH, Atolagbe OO, Cooke DT. The use of the international classification of diseases, tenth revision, clinical modification and procedure classification system in clinical and health services research: the devil is in the details. JAMA Surg 2019;154:1089–90. 31553423 10.1001/jamasurg.2019.2899

[43] Zack T, Lehman E, Suzgun M, Rodriguez JA, Celi LA, Gichoya J, Assessing the potential of GPT-4 to perpetuate racial and gender biases in health care: a model evaluation study. The Lancet Digit Health 2024;6:e12–2. 38123252 10.1016/S2589-7500(23)00225-X

[44] Nastasi AJ, Courtright KR, Halpern SD, Weissman GE. A vignette-based evaluation of ChatGPT’s ability to provide appropriate and equitable medical advice across care contexts. Sci Rep 2023;13:17885–6. 37857839 10.1038/s41598-023-45223-y PMC10587094

[45] Chakravorty S, Williams TN. Sickle cell disease: a neglected chronic disease of increasing global health importance. Arch Dis Child 2015;100:48–53. 25239949 10.1136/archdischild-2013-303773 PMC4285890

[46] White NJ, Watson JA, Uyoga S, Williams TN, Maitland KM. Substantial misdiagnosis of severe malaria in African children. The Lancet 2022;400:807–8. 10.1016/S0140-6736(22)01600-2 36088944

[47] Stewart C, Pepper MS. Cystic fibrosis on the African continent. Genet Med 2016;18:653–62. 26656651 10.1038/gim.2015.157

[48] Ronicke S, Hirsch MC, Türk E, Larionov K, Tientcheu D, Wagner AD. Can a decision support system accelerate rare disease diagnosis? Evaluating the potential impact of Ada DX in a retrospective study. Orphanet J Rare Dis 2019;14:69–70. 30898118 10.1186/s13023-019-1040-6 PMC6427854

[49] Adler-Milstein J, Redelmeier DA, Wachter RM. The limits of clinician vigilance as an AI safety bulwark. JAMA 2024;331:1172–4. 10.1001/jama.2024.3620 38483397

[50] Ayers JW, Desai N, Smith DM. Regulate artificial intelligence in health care by prioritizing patient outcomes. JAMA 2024;331:639–40. 38285467 10.1001/jama.2024.0549

[51] Dembek Z, Hadeed S, Tigabu B, Schwartz-Watjen K, Glass M, Dressner M, Ebola virus disease outbreaks: lessons learned from past and facing future challenges. Mil Med 2024;189:e1470–8. 38743575 10.1093/milmed/usae204

